# Colorado as a Health Resort

**Published:** 1874-01-15

**Authors:** Charles Denison


### COLORADO AS A HEALTH RESORT.

By Charles Denison, M.D.

To the Editors of the Medical Examiner: The following are some of my first *impressions* of this climate, which you wished me to send you. Notwithstanding the usual belief—which is like the old adage, "A change of pastures makes fat calves,"—that a change of climate and surroundings of most any kind is usually beneficial to the health of chronic invalids, nevertheless, a residence in this bracing climate has a peculiarly revivifying influence on most all phthisical patients who come here quite early in the progress of their disease. Dyspepsia, too, and most all ailments due to mal-nutrition, are usually banished, or very much relieved, by a sojourn here. But the salutary influence of this rarified atmosphere is best shown in the relief of asthma. It is said that few asthmatics have come here without permanent or marked benefit, the chief exceptions being those who have organic disease of the heart, or marked emphysema. Not long since, the asthmatics held a convention in Denver. The object was to gather their evidence, that those similarly afflicted throughout the States might be benefited thereby. It was a commendable undertaking, and quite successful. Over a hundred cases were tabulated of those who had found entire or decided relief in Colorado. Many had suffered for a long time, and some intensely, unable even to lie down for months at a time. The report of the proceedings of this convention is to be printed, and probably will be cheerfully sent to any interested, by Mr. F. J. B. Crane, Denver, Colorado. Mr. Crane is an estimable gentleman, who had been a sufferer from asthma many years, and has taken a prominent part in this movement.

The influences which seem to combine, in Colorado, to benefit sufferers from thoracic diseases, are various. Probably more important than is usually recognized, is *altitude*, which, it would seem, is chiefly mechanical in its effect, for consequent upon it there is lessened atmospheric pressure, and it is positively necessary for lungs to have a proportionately greater expansion, in order to get the needed amount of oxygen, than on the coast, five thousand three hundred feet below Denver.

The circulation, of course, acts in harmony with this increased respiratory power, which results in increased combustion. Waste and repair are both more rapid and complete; adipose is called into requisition; and animal economy becomes dominant. The average rancheman of Colorado gives one an idea of the effect of outdoor life in elevated regions. He is a *lean* fellow, with well-browned complexion, good hard muscles, and great endurance. The idea of such a man having tuberculosis would seem almost preposterous. His blood is habitually well oxygenized; and even if he had tubercular matter in him to deposit, the room could hardly be spared for it in his lungs. He may get pneumonia, perhaps, resulting in gangrene or pneumonic phthisis; but it seems the origination of miliary tubercle rarely occurs above the altitude of five thousand feet. The increased amount of electricity, too, due to altitude, very likely has a salutary influence in strengthening enfeebled nervous systems.

The small amount of rain-fall (about twelve inches a year), the great amount of sunshine, and the porosity of the soil, favor a dryness of the atmosphere directly opposite to what Dr. Bowditch, of Boston, has proved, by tabulated opinions of physicians, to be a chief cause of phthisis —"soil moisture."

At the foot of the eastern slope of the Rocky Mountains, extending north and south, is a belt of land, say thirty miles wide, in the middle of which, toward the north, is Denver. This, including the valleys and parks among the mountains, will probably be the tillable portion of Colorado, because, as irrigation is necessary for the cultivation of most products, on account of the aridity of the soil, this land may "be brought *under ditch*" from the streams coming down from among the snow-capped mountains. The range is about a hundred miles wide in Central Colorado; and it is among these lofty mountains that the clouds are drained of thirty to forty inches of rain, or melted snow, in a year. Most of the latter remains to be melted by the summer's sun, at just the season when water is of the most use to the farmer for irrigation.

The soil is a deep, sandy loam, made up of the washings—the debris of centuries—from this vast mountain region, and seems to contain the chemicals, especially alkalies, for producing cereals of remarkable richness.

The wheat of Colorado is acknowledged to be unsurpassed by any in America. The beef of this country, from cattle who pick their own living all the year round from the short prairie-grass covering these broad, arid plains, is much better, and the milk much richer, than one would at first suppose. In addition to the above, venison, antelope, other spoils of the hunter, and a good market, afford variety in edibles.

The temperature of this part of Colorado in winter, averages about the same as that in New England, southern, central, and northern Ohio, Indiana and Illinois; but it does not *seem* so cold, because of the dryness of the air, the little amount of snow, and the usually sunny days, the nights, when people are indoors, being generally cold. The diurnal variations in temperature are considerable, and quite regular, the difference between that at 2 P.M. and that at 7 A.M. or 9 P.M. averaging fifteen to twenty degrees.

The following is the weather report for the year 1873, as furnished by Lieutenant Henry Fenton, United States Signal Officer, stationed at Denver:

| | Temperature. | | | Rain and Melted Snow. |
|---|---|---|---|---|
| Months. | Highest | Lowest | Mean. | |
| January | 62 | —17 | 30 | .13 |
| February | 60 | 3 | 30 | .24 |
| March | 75 | 11 | 40 | .22 |
| April | 81 | 10 | 40 | 2.43 |
| May | 84 | 27 | 63 | .75 |
| June | 99 | 48 | 71 | 2.24 |
| July | 99 | 42 | 71 | 2.00 |
| August | 96 | 50 | 71 | 1.41 |
| September | 90 | 28 | 60 | .89 |
| October | 86 | 1 | 45 | .73 |
| November | 71 | —1 | 41 | .16 |
| December | 59 | —5 | 22 | .53 |
| Mean for the year | 99 | —17 | 48 | 11.73 |

During the past two months there have been five quite disagreeable, stormy days, nearly all the rest of the days being sunny and genial. The wind has not blown much of the time, yet occasionally it gets up quite a commotion, constituting what are called "wind-storms," perhaps more properly, *dust-storms.* There have been two of these during the past two months, of from two to four hours' duration. One of these occurred in the night, much to the discomfort of some strangers, who had a room in the third story of a hotel. The gentleman and his wife came down at 3 A.M., preferring not to "run the risk of having the walls blown in upon them."

The diseases prevalent in, and probably incident to, great altitudes, in winter, seem to be catarrh, which annoys many new comers, rheumatism, and bronchitis.

The change of residence from lowlands to this airy region is of such a nature that an accurate knowledge of the character and varying influences of this climate is of special importance to those suffering with disease of the respiratory or circulatory organs. This will be evident to any one who will impartially study the results of residence here of various classes of invalids, especially *consumptives;* and thus it appears that a more careful discrimination should be made than has hitherto obtained as to who should, and who should not, resort to this climate and altitude.

From what I have written above as to the *mechanical* effect of altitude, it would appear, on reflection, that this element might prove injurious as well as, and if not, beneficial; that to some, who hardly had lung surface enough to get a fair amount of oxygen below, the rarified atmosphere of even the lower portions of Colorado might not be the most appropriate. If the evidence of *probable* deaths from *altitude* were needed to substantiate this statement, it could be given. Among others, the case of John C. Heenan, the pugilist, would seem to be interesting in this connection. Once he received a blow on the left side of the upper part of his chest, from the effects of which he never wholly recovered. In Colorado, he had an attack of hæmorrhage. After a while, apparently in pretty good condition, he started for Southern California. He is said to have died of hæmorrhage, in the cars, at one of the highest points on the Union Pacific railroad.

Those who have never visited our mountain region (ranging as it does from six to fourteen thousand feet above the sea), in advising their patients *to go up into the mountains in Colorado, and have a good time,* would seem to act unadvisedly, in view of the hæmorrhages and other drawbacks such a course is said to have entailed.

Generally speaking, patients, on coming here, do well to take life easy for awhile, till they get somewhat acclimated: not to allow the buoyancy and exhilaration of this light air to deceive them into too great confidence in their powers of endurance, till the circulation and respiration get used to the change.

As to this immediate vicinity (our personal experience has not yet extended farther in Colorado), when we think of the multitude who are insiduously gliding into confirmed phthisis in low, damp homes in the

States, who could have almost certainly been restored to health; and of the many who come too late to be benefited *here*, to be hastened to their graves, or sent back East, disappointed, the words of Burns seem appropriate:

> "It's hardly in a body's power
> To keep, at times, frae bein' sour,
> To see how things are shared."

And yet it is very difficult to give a rule which should decide, in all cases, who should come as high as this, and the method of their coming. But were I to decide, in a general way, who should *not* come directly here, feeling that they could much better go elsewhere, at least in winter, I should name the following classes of cases: Phthisis pulmonalis, complicated with organic disease of the heart; phthisical patients, one-fourth or more of whose lungs are seriously diseased, or rendered useless, especially if they contain any cavities; if in patients of decidedly hæmorrhagic diathesis, or those whose pulse is uniformly very rapid; or, if in women, or patients of delicate constitutions, whose lungs are quite liable to take on frequently recurring attacks of inflammation.

As March and April are said to be "winter-months" here, as well as in many places East, perhaps the time specified above ought to be extended toward May. Even in summer, considering the imperative demand, with each decided elevation, for increased expansion of the lungs, or breathing capacity, it would seem that a *sudden* change from below a thousand to over five thousand feet above the sea would generally be an extreme measure for such as I have specified; and this, too, notwithstanding it is said there are those living hereabouts who once had cavities in their lungs, and in whom the disease seems now to be arrested.

Some of the most remarkable instances of the arrest of phthisis have been in those who came up by degrees, in wagons, through Kansas; and it may be that the ratio of deaths, to all deaths from this disease in Colorado, would be very much lessened, if all patients in coming to the lower Rocky Mountain regions resorted to the old-fashioned means of travel by *ox-team* across the Plains.

From personal experience, the effect of the sudden rise coming through Kansas seemed quite decided to one who had had, nearly a year before, pulmonary hæmorrhage, followed by chronic pneumonia, and probably partial closure of some peripheral portions of lung. This was evinced, *at first*, by quite marked increase in the force and rapidity of the heart's action, and increased respiratory activity, with a most disagreeable desire for *more air*. This acceleration of breathing and circulation is followed, shortly after arriving here, by a congested feeling in the chest, with an increased tendency to hæmorrhage for awhile, which, however, passes off with the hurried breathing; and improvement in both weight and muscle continues for several weeks.

As we came up on the Kansas Pacific Railway, over four thousand feet in twenty-four hours, an idea suggested itself that *expiration* decreased proportionately to the increase in altitude, while inspiration remained about the same. This—to me, at least, a discovery, which, in some unexplainable way, as affecting *prolonged* expiration, might, perhaps, have something to do with the arrest of phthisis—led to an invention. It was an instrument to measure the expiratory and inspiratory movements of the chest, as the sphygmograph measures the pulse. The stable portions of the apparatus were to rest on the hips and shoulders, and respiration was to be measured by levers, one end of each lever resting on the chest, and the other on paper moved by clock-work.

It seems that such an instrument might be of much value in the diagnosis, and, perhaps, in the prognosis of phthisis. However, I fear my idea came a little late, for the *Medical and Surgical Reporter* of December 6th gave a description of a similar instrument, called the stethograph, lately " devised by Dr. F. Riegel."

Much might be written about other conditions besides altitude, which are of special importance to the medical profession, and invalids recommended to this country, such as temperature, various localities, occupation, social surroundings, home-comforts, etc.; but this, the limits of the present article will hardly allow.

It was intended only to touch upon the salient points; and the writer will be well pleased, if, in this paper, he has even poorly shown some of the peculiar advantages to health-seekers which obtain in Colorado, or if, in any way, he has aided physicians in appreciating the importance of a more comprehensive and thorough study than has yet been carried out, of all our various climatic influences in the United States and Territories.

Denver, Colorado, Dec. 31, 1873.

